# Hypophosphatemic osteomalacia caused by diffuse tenosynovial giant cell tumor misdiagnosed as spondyloarthritis: a case report

**DOI:** 10.3389/fendo.2026.1808434

**Published:** 2026-07-13

**Authors:** Yazhen Su, Xingxing Zhao, Ruihong Hou, Dan Ma, Ke Xu, Liyun Zhang

**Affiliations:** 1 Department of Rheumatology and Immunology, Shanxi Bethune Hospital, Shanxi Academy of Medical Sciences, Tongji Shanxi Hospital, Taiyuan, China; 2 Shanxi Province Clinical Research Center for Dermatologic and Immunologic Diseases (Rheumatic diseases), Taiyuan, China

**Keywords:** case report, low back pain, spondyloarthritis (SpA), tenosynovial giant cell tumor (TGCT), tumor-induced osteomalacia (TIO)

## Abstract

Tumor-induced osteomalacia (TIO) is an acquired hypophosphatemic osteodystrophy caused by increased renal phosphate excretion due to tumors. Tenosynovial giant cell tumor (TGCT) is a benign mesenchymal tumor but rarely causes hypophosphatemia. TIO is a clinically rare disease, and its clinical manifestations often overlap with rheumatic diseases such as spondyloarthritis (SpA), leading to misdiagnosis. A 32-year-old female had a prolonged misdiagnosis of undifferentiated spondyloarthritis due to chronic low back pain, limb weakness, and spinal deformity, with poor response to non-steroidal anti-inflammatory drugs and various biologic agents. After hospitalization, a systematic biochemical screening for metabolic bone disease revealed decreased serum phosphate, calcium, and 25-hydroxyvitamin D levels, along with elevated alkaline phosphatase. Subsequently, a previously undetected tumor in the right plantar region was successfully localized using 68Ga DOTA-TATE PET/CT. After complete surgical removal of the tumor, the patient’s serum phosphate levels rapidly returned to normal, and bone pain symptoms were significantly alleviated. After histopathological staining of the tumor, it was considered to be diffuse tenosynovial giant cell tumor (D-TGCT). This case clearly demonstrates a TIO caused by the rare disease D-TGCT. Its diagnosis and treatment journey not only reveals the clinical relevance of D-TGCT as an atypical pathogenic tumor but also highlights the critical importance of multidisciplinary collaboration and advanced diagnostic techniques in tackling such rare diseases. This provides clear guidance for enhancing clinical awareness and optimizing treatment strategies.

## Introduction

TIO is a rare paraneoplastic syndrome characterized by the excessive secretion of fibroblast growth factor 23(FGF23) by tumors (1). This leads to increased renal phosphate excretion and suppressed active vitamin D production, which subsequently cause hypophosphatemia, bone mineralization disorders, and clinical manifestations such as progressive bone pain, muscle weakness, and pathological fractures (2). The most common type of tumor associated with this disease is phosphaturic mesenchymal tumor (PMT), accounting for approximately 65.4% of cases (3). TGCT, as a benign fibrous tissue tumor primarily affecting joint synovium, bursae, or tendon sheaths, has rarely been reported as a cause of TIO (4, 5). The initial symptoms of TIO, such as chronic low back pain, morning stiffness, peripheral joint discomfort, and possible imaging changes in the sacroiliac joints, significantly overlap with rheumatic diseases like SpA. Moreover, the pathogenic tumors are usually small, slow-growing, and located in hidden areas, making clinical diagnosis extremely challenging. This results in a high misdiagnosis rate, and patients often experience a long and tortuous journey to seek medical attention.

This case report reveals a rare and easily overlooked causal association—TIO caused by D-TGCT. It emphasizes the importance of conducting systematic metabolic bone disease screening in patients presenting with “inflammatory” back pain who do not respond to conventional anti-inflammatory treatment. Additionally, it highlights the excellent sensitivity of functional molecular imaging, such as 68Ga-DOTA-TATE PET/CT, for locating hidden tumors.

## Case description

The patient is a 32-year-old female who presented with low back pain and limb weakness for 5 years, worsening with spinal deformity for 2 years. Five years prior to the definitive diagnosis in the present admission, the patient developed low back pain without obvious inducement, unrelated to activity, and gradually experienced nighttime pain and difficulty turning over, accompanied by morning stiffness, with little relief after activity, which was not taken seriously. Subsequently, the patient experienced limb weakness, particularly in the lower limbs, with a subjective decrease in walking distance, unsteadiness while walking, and changes in gait, unable to go upstairs or squat, with acupuncture and other treatments showing poor efficacy. Three years preceding diagnosis, the patient’s above symptoms gradually worsened, accompanied by bilateral thigh root pain, so she went to the local hospital’s neurology department for consultation. Relevant examinations showed no abnormalities, and auto-antibodies were negative, thus no clear diagnosis or treatment was made. The same year, she visited the rheumatology and immunology department. Following relevant examinations, sacroiliac joint CT revealed bone destruction, leading to a diagnosis of “undifferentiated SpA.” She was treated with oral non-steroidal anti-inflammatory drugs, followed by sequential regular administration of recombinant human TNF receptor-antibody fusion protein and adalimumab for six months; however, the treatment outcomes remained unsatisfactory. Two years preceding diagnosis, the patient’s above symptoms gradually worsened, and spinal deformity occurred, accompanied by limited limb movement and a height reduction of 10 cm. The patient has visited the Departments of Rheumatology and Immunology, and Orthopedics at multiple hospitals, receiving symptomatic treatment with unsatisfactory results. After multiple previous outpatient visits at external hospitals, she was admitted to our Department of Rheumatology and Immunology in July 2024 for further definitive evaluation and treatment. Throughout the disease course, there were no rashes, photosensitivity, oral ulcers, erythema nodosum, nor any color changes in examination showed the hands upon exposure to cold. There were no special past or family histories.

Physical examination: Pulse 90 beats per minute, blood pressure 109/84 mmHg. The patient was unable to stand upright owing to severe spinal deformity and intractable generalized bone pain, and hence standing height and body weight could not be measured. The integument was intact without café-au-lait macules. The patient’s normal spinal physiological curvature had disappeared, with thoracic kyphosis deformity and limited three-dimensional movements. Tenderness was present in each vertebra and paravertebral muscles. There was no joint swelling or tenderness, but the patient was unable to perform or coordinate the bilateral “4” test. Muscle strength was graded 5^-^ on manual muscle testing, muscle tone was normal, and there was no edema in either lower limb.

Laboratory tests (all venous blood samples were collected after an overnight fast): Inflammatory and serologic markers: ESR 3 mm/h (reference for adult females:<20 mm/h), CRP<1.00 mg/L (0.00–8.00 mg/L), HLA-B27 (-); other inflammatory biomarkers and autoantibodies were unremarkable. Bone metabolism indicators: parathyroid hormone (PTH) 59.08 pg/mL (15–65 pg/mL), alkaline phosphatase (ALP) 234.7 IU/L (30–100 IU/L), serum creatinine 42.0 μmol/L (44-97 μmol/L for females), blood urea nitrogen 2.4 mmol/L (2.9-8.2 mmol/L). Serum electrolytes: potassium 3.43 mmol/L (3.5-5.5 mmol/L), inorganic phosphorus 0.56 mmol/L (0.81-1.45 mmol/L), total calcium 2.10 mmol/L (2.20-2.65 mmol/L). 25-hydroxyvitamin D: 17.55 ng/mL (30–100 ng/mL). Twenty-four-hour urinary quantification: total urine volume 1.5 L with urinary phosphorus 15.78 mmol/24 h (12.9-42.0 mmol/24 h). A repeat 24 h urine test performed 16 days later showed urinary phosphorus of 29.58 mmol/24 h.

**Figure 1:**
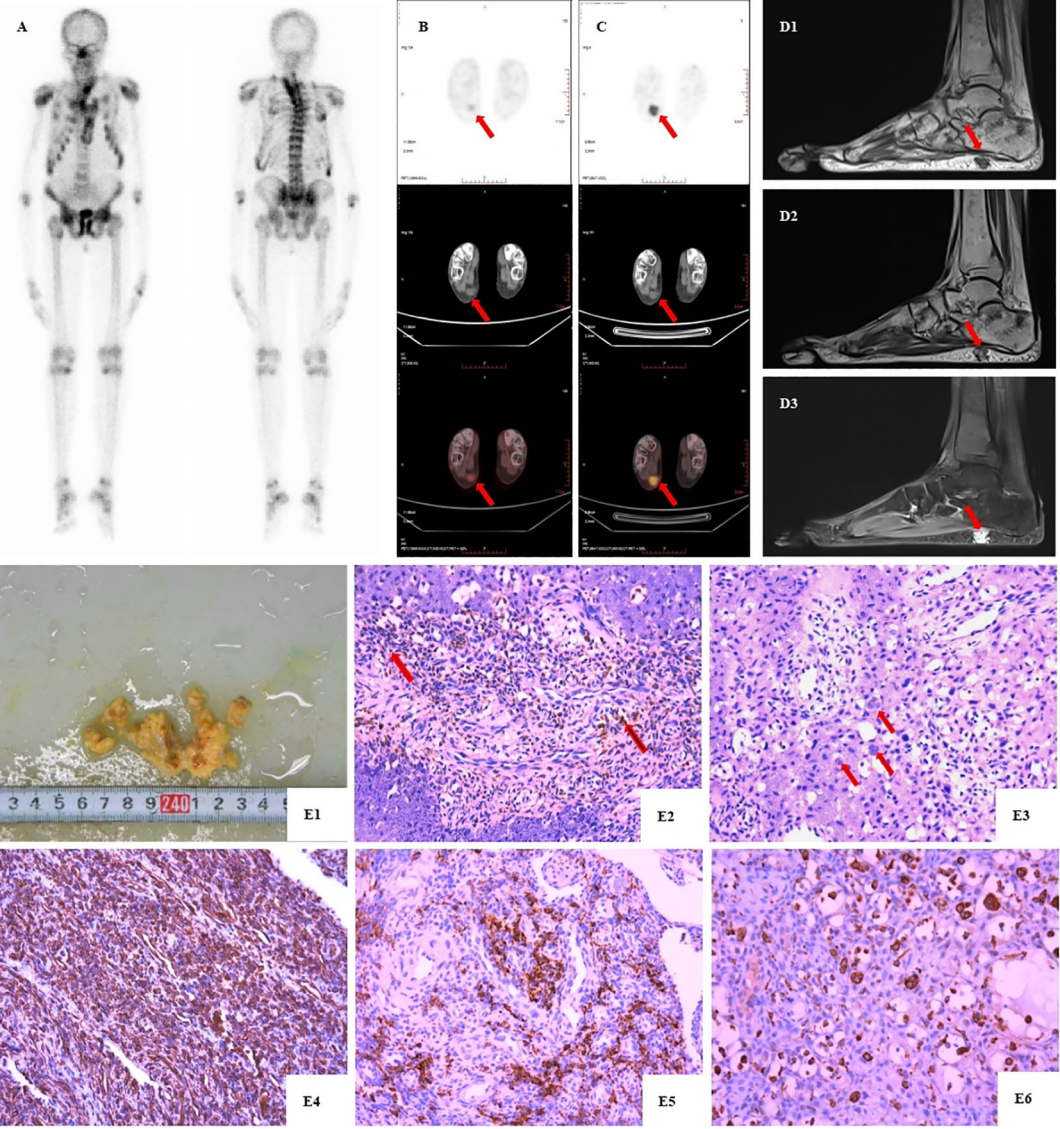
**(A)** Whole body bone scan showed increased multi bone metabolism with changes in bone density, and multiple old fractures.; **(B)** PET-CT showed a subcutaneous nodule in the right sole, with mild increase in FDG metabolism (SUVmax 1.74); **(C)** ^68^Ga-DOTA-TATE PET/CT showed highly metabolized of the nodule (SUVmax 5.31), with a size of approximately 1.2*1.1cm.; **(D)** Right foot MRI showed a long T1 (D1) and equal T2 (D2) signal shadow, the fat suppression sequence is high signal (D3), with a range of about 1.3*2.52*1.23cm in the subcutaneous fat layer near the heel; **(E)** Specimens were solid gray yellow and gray brown nodules (E1); HE staining shows deposition of hemosiderin (E2) and multinucleated giant cells (E3); Positive expression of Vimentin staining (E4); CD163 staining (E5); CD68 staining (E6).

Imaging findings: Spinal radiographs revealed scoliosis deformity, thoracic kyphosis, flattening of partial thoracic vertebral bodies, and diffuse reduction in vertebral bone density. Pelvic radiographs showed pelvic deformity accompanied by decreased bone mineral density, irregular contours of bilateral inferior pubic rami, and transverse linear hypodense lesions in the proximal bilateral femurs, suggestive of a metabolic bone disorder. Following intravenous administration of 30 mCi ^99m^Tc-MDP, whole-body SPECT/CT was acquired 3 hours post-injection. Diffusely increased radiotracer uptake was observed throughout multiple vertebral bodies, ribs, pelvic bones, bilateral shoulders, elbows, hips as well as hand and foot joints. Corresponding CT reformats demonstrated generalized cortical thinning, distorted vertebral contour, thoracic cage malformation, and focal disruption of cortical continuity in partial ribs, consistent with a metabolic bone disorder complicated by multiple fractures (**Figure 1A**). Laboratory bone turnover markers indicated enhanced bone resorption and bone formation. Pelvic CT targeting the sacroiliac joints revealed mild bone marrow edema confined to the bilateral inferior pubic rami and acetabula; no particular surface erosion, periarticular sclerosis, fatty marrow infiltration or sacroiliac ankylosis was identified. Considering the patient’s medical history, symptoms and signs, the possibility of a diagnosis of TIO is high. To further elucidate the etiology, the patient underwent a PET/CT scan (**Figures 1B, C**), which showed a subcutaneous nodule in the right plantar region near the calcaneus with mildly increased FDG metabolism; the maximum standardized uptake value (SUVmax) was approximately 1.74. This nodule demonstrated high metabolic activity on the 68Ga DOTA-TATE PET/CT, with a SUVmax of approximately 5.31, measuring approximately 1.2 × 1.1 cm. A solid hypoechoic mass is visible on local ultrasound, and MRI reveals a mass-like signal shadow (**Figure 1D**). After orthopedic consultation, the patient underwent surgical removal of the nodule.

The postoperative pathological results report (**Figure 1E**): HE staining shows hemosiderin-laden macrophages (E2) and multinucleated giant cells (E3); immunohistochemistry shows positive staining for vimentin (E4), CD163 (E5) and CD68 (E6). Therefore, the nodule is diagnosed as D-TGCT, and the patient was ultimately diagnosed with TIO caused by D-TGCT.

**Figure 2:**
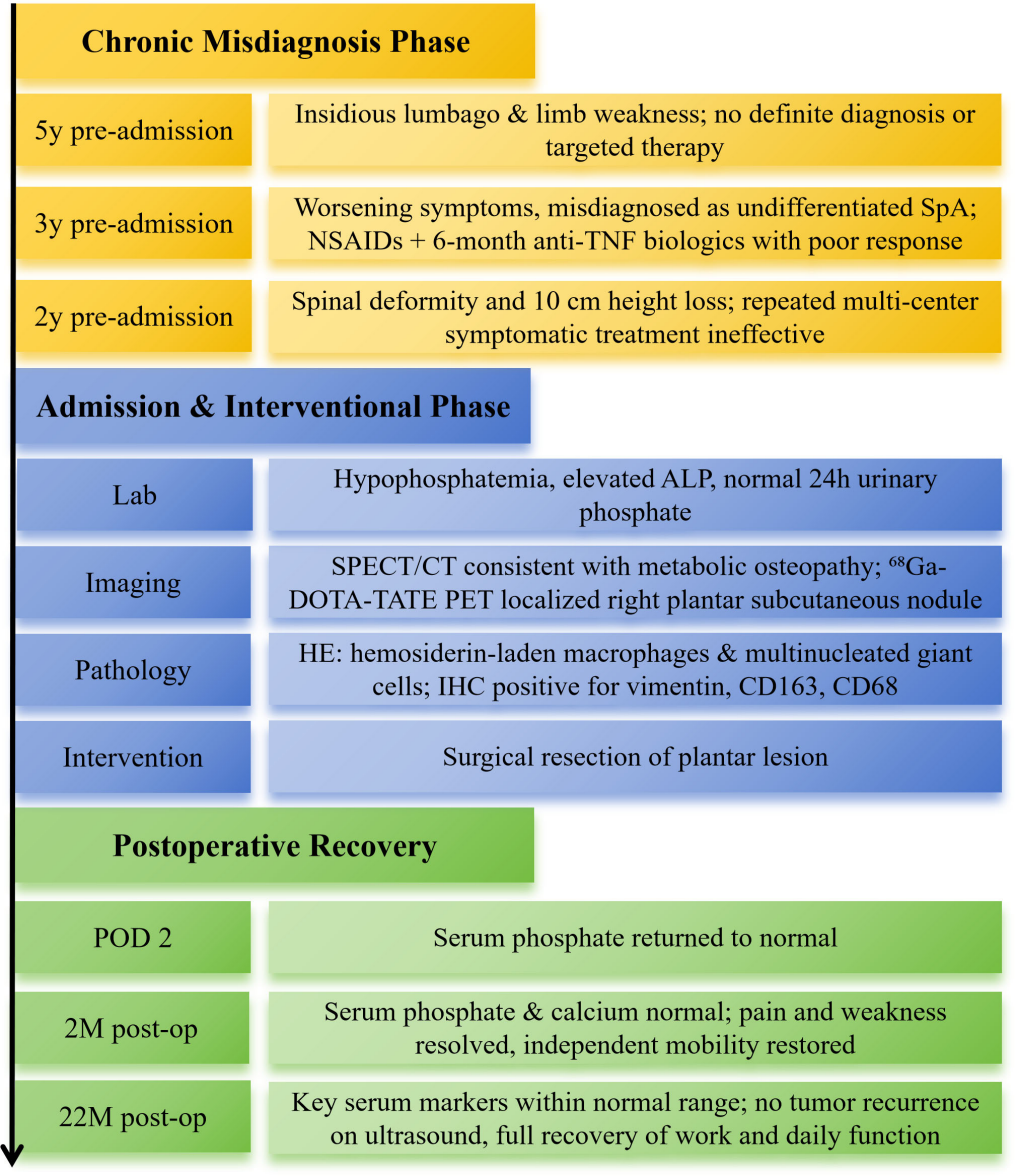
It summarizes the timeline of TIO caused by D-TGCT patients, including the onset of symptoms, long-term misdiagnosis, ineffective treatment, tumor localization, surgical resection, and prognosis.

On postoperative day 2, the patient’s fasting serum phosphorus recovered to 0.87 mmol/L (reference range: 0.81–1.45 mmol/L). One month after surgery, diffuse skeletal pain was markedly alleviated compared with the preoperative state. Re-examination at 2 months postoperatively showed that fasting serum phosphorus and serum calcium were within normal limits; the patient’s generalized bone pain and limb weakness were substantially relieved, and she regained full capacity for independent daily living. Lumbar bone mineral density testing performed at 7 months postoperatively revealed no obvious abnormalities in lumbar bone mineral content. During follow-up, the patient took oral calcium supplements as instructed, maintained a balanced diet, and performed progressive rehabilitation exercises within her physical tolerance. At the 22-month postoperative follow-up visit, the patient underwent comprehensive re-evaluation: fasting serum phosphorus, serum calcium and alkaline phosphatase all fell within normal reference ranges, and ultrasound of the right plantar region showed no definite signs of tumor recurrence. At present, the patient has fully returned to regular work and daily activities, and has agreed to continue long-term regular follow-up monitoring. The detailed chronological progression of symptoms, misdiagnosis, treatment and postoperative recovery is summarized in **Figure 2**.

## Discussion

Tumor-induced osteomalacia (TIO) often presents with non-specific manifestations including progressive bone pain, muscle weakness, gait difficulty and multiple fractures. Some patients complain of low back pain, morning stiffness and peripheral joint pain, accompanied by abnormal imaging findings of the sacroiliac joints. Such clinical manifestations are highly overlapping with those of rheumatic diseases, metabolic bone diseases and primary myopathies, which can easily mislead clinical diagnosis. In this case, the patient initially presented with low back pain, limb weakness, and morning stiffness. The sacroiliac joint CT indicated “bone destruction” resembling SpA, which led to a misdiagnosis of “undifferentiated SpA”. However, sacroiliac CT performed in our institution only demonstrated peri-acetabular and peripubic bone marrow edema without pathognomonic osseous destruction. The previous tentative diagnosis of undifferentiated SpA was likely attributable to misinterpreting isolated marrow edema as true bone destruction, which constituted the core reason for initial misdiagnosis.

Epidemiological data worldwide consistently reveals substantial diagnostic delay in patients with TIO from symptom onset to definitive confirmation. A retrospective cohort enrolling 144 TIO cases reported a mean interval of 2.9 ± 2.3 years from symptom onset to diagnosis and 5.4 ± 4.2 years from initial symptoms to curative tumorectomy, with an overall misdiagnosis rate up to 95.1%; spondyloarthritis ranks among the most frequent erroneous diagnoses (6). A single-center study documented an average diagnostic delay of approximately 4.9 years with wide inter-individual variability (7). The 5-year span between disease onset and surgical confirmation in our patient aligns well with these published epidemiological findings (8–10).

The diagnosis of TIO relies on characteristic biochemical abnormalities and imaging localization techniques. Typical biochemical manifestations consist of hypophosphatemia, inadequately preserved urinary phosphate excretion without compensatory reduction, low or normal serum 1,25-dihydroxyvitamin D [1,25(OH)_2_D], and elevated FGF23 (11). Restricted by objective clinical conditions, measurements of serum FGF23, 1,25-(OH)_2_D and tubular maximum reabsorption of phosphate per glomerular filtration rate (TmP/GFR) were unavailable in the present case. The decrease in the patient’s blood calcium and 25-hydroxyvitamin D is considered to be caused by long-term excessive consumption of bone mineralization. Combined with the absence of a history of insufficient nutritional intake or gastrointestinal absorption disorders, nutritional osteomalacia can be largely ruled out. Radiographically, TIO may present as osteoporosis or pseudofractures (such as Looser’s zones), but these imaging findings lack specificity. Most causative tumors of TIO are PMTs, which express somatostatin receptor type 2 (SSTR2). However, these lesions are typically small in volume with low receptor density, rendering tumor localization diagnostically challenging and necessitating a combination of multiple imaging modalities. On CT and MRI, lesions may present as osteolytic, sclerotic, or mixed changes, yet such manifestations are highly heterogeneous and non-specific (12). Fundamentally, conventional CT and MRI are morphological imaging modalities that rely on detectable anatomical structural alterations. Early-stage TIO-associated tumors often do not induce evident bone destruction or form distinguishable soft tissue masses, resulting in a high false-negative rate. Furthermore, TIO-causing tumors frequently arise in anatomically complex regions such as the tendinous sheaths of the extremities and craniofacial bones, and lack characteristic imaging features. Small lesions in these specialized sites are easily missed on MRI, which accounts for the core reason why multiple prior local MRI scans failed to identify the foot tumor in our patient. Indium-111 pentetreotide scintigraphy, the first-generation somatostatin receptor imaging modality, suffers from insufficient SSTR2 binding affinity and limited spatial resolution (13, 14). It exhibits relatively low sensitivity for TIO tumor localization with suboptimal detection efficiency and a high risk of false-negative results (13, 14). In contrast, ^68^Ga-DOTATATE PET/CT and [¹^8^F]F-OC PET/CT demonstrate high binding affinity for SSTR2, enabling the detection of subcentimeter lesions (<1 cm) with superior sensitivity and effective differentiation between neoplastic lesions and inflammatory changes. These modalities are currently internationally recognized as the first-line imaging approaches for TIO localization (13–17). For patients with negative imaging findings, selective venous sampling for FGF23 gradient measurement serves as a complementary tool to regionalize the occult tumor (18). In our case, the patient experienced a 5-year diagnostic delay, and the causative tumor was ultimately successfully localized via ^68^Ga-DOTATATE PET/CT, which further underscores the clinical guiding value of this case report.

Histopathological examination is the gold standard for the diagnosis of pathogenic tumors. PMT presents as morphologically diverse mesenchymal tumors, and immunohistochemical staining demonstrates positivity for FGF23 (19). Although PMT is the most common cause of TIO, the spectrum of pathogenic tumors for TIO is not limited to PMT. The final pathological diagnosis in this case was D-TGCT, which is a very rare cause of TIO. D-TGCT, also known as pigmented villonodular synovitis (PVNS), is a locally aggressive benign tumor that appears as ill-defined, diffusely growing multiple lesions on MRI (5, 20). It commonly occurs in weight-bearing joints such as the knee and hip and has a relatively high recurrence rate (5, 20). Its histopathological features include multinucleated giant cells, foamy macrophages, and hemosiderin-laden macrophage infiltration, and immunohistochemical staining demonstrates positivity for macrophage markers such as CD68 and CD163 (21).

The biochemical examination of this patient showed decreased serum phosphate, serum calcium, and elevated ALP, consistent with the biochemical characteristics of TIO. PET/CT examination revealed a subcutaneous nodule on the plantar aspect of the right foot near the calcaneus with high metabolic activity. Postoperative pathological examination of the nodule showed hemosiderin-laden macrophages and multinucleated giant cells, with immunohistochemical positivity for vimentin, CD68, and CD163, consistent with the diagnosis of D-TGCT. We systematically investigated the core etiological spectrum of hypophosphatemic osteomalacia, including genetic diseases (e.g., X-linked hypophosphatemic rickets, autosomal dominant hypophosphatemic rickets), systemic diseases (e.g., Fanconi syndrome), drug factors (e.g., adefovir dipivoxil), and tumor-related factors (22, 23). Given the patient’s young age of onset, absence of family history, no history of liver disease or specific medication use, and no clinical manifestations of Fanconi syndrome, tumor-induced hypophosphatemic osteomalacia is the most likely etiology.

In summary, this case is primarily considered as D-TGCT-induced hypophosphatemic osteomalacia, based on the following indirect clinical evidence: typical clinical manifestations of hypophosphatemic osteomalacia, radiological features of systemic metabolic bone disease, pathologically confirmed D-TGCT lesions, and the characteristic outcome of rapid normalization of serum phosphorus after lesion resection. A substantial body of evidence-based medicine indicates that rapid normalization of serum phosphorus within 48–72 hours after complete tumor resection is the most specific clinical hallmark of TIO and an important validation criterion for diagnosis (24–27). In this patient, serum phosphorus normalized on the second postoperative day, consistent with the typical outcome reported in the literature, further supporting the rationality of the above clinical diagnosis. Due to limitations in hospital testing conditions, immunohistochemistry for FGF23 and molecular biological testing of tumor tissue were not performed, resulting in a lack of direct molecular biological evidence. However, based on the series of clinical laboratory tests, imaging features, and rapid improvement in postoperative biochemical indicators, the above clinical diagnosis is established.

Surgical resection is the preferred and curative treatment method for TIO, with an efficacy rate of over 90%. Patients’ biochemical indicators usually return to normal within 2–7 days after surgery, and symptoms such as bone pain can be significantly relieved within one month (28, 29), which is completely consistent with the clinical outcome of the present case. For patients with TIO in whom the causative tumor cannot be localized or is deemed unresectable, image-guided ablation therapy (e.g., cryoablation) (30) or pharmacotherapy (e.g., burosumab) may be considered as alternative treatment strategies. Burosumab is a fully human monoclonal antibody targeting FGF23, and it is particularly indicated for TIO patients with elevated circulating FGF23 levels (31). Accumulating clinical evidence has demonstrated that burosumab effectively normalizes serum phosphorus concentrations and promotes fracture healing in affected individuals (31, 32). In terms of prognosis, most pathogenic tumors (including PMT and D-TGCT) are benign, with favorable outcomes after surgical resection. However, there are a few malignant PMTs with higher FGF23 levels, which increase the risk of death, necessitating long-term follow-up to monitor recurrence and metastasis (2).

The diagnostic journey of this case profoundly reveals the diagnostic pitfalls between TIO and SpA. Both can present as chronic low back pain, morning stiffness, and peripheral joint symptoms. They may also show imaging abnormalities of the sacroiliac joint (such as the misinterpreted “bone destruction” in this case), which makes them easily confused. However, there are essential differences in the pathological mechanisms and core characteristics between the two disorders. SpA is an inflammatory disease characterized by inflammatory back pain, sacroiliitis, and HLA-B27 positivity. Late-stage imaging findings may include sacroiliac joint fusion or ligamentous bone spurs. Laboratory tests may show elevated inflammatory markers, but there are no biochemical abnormalities specific to TIO (such as hypophosphatemia) (33). In contrast, TIO is a paraneoplastic disease, with the core feature being renal hypophosphatemia accompanied by elevated FGF23 levels, and inflammatory markers are not specifically elevated (13). The key distinguishing point is that TIO has the aforementioned characteristic biochemical abnormalities, and the diagnosis can ultimately be confirmed through tumor location. Clinicians must consider metabolic bone diseases such as TIO in the differential diagnosis when faced with atypical “inflammatory” musculoskeletal pain, especially in patients who do not respond to conventional anti-inflammatory treatment. Timely systematic biochemical evaluations are essential to avoid irreversible skeletal damage (such as severe height loss and spinal deformities) caused by long-term misdiagnosis.

## Limitations

This study has several objective limitations. First, the patient presented with persistent hypophosphatemia accompanied by a lack of compensatory reduction in urinary phosphate excretion, a clinical manifestation strongly suggestive of renal phosphate wasting. However, simultaneous urinary creatinine was not measured, so the TmP/GFR could not be calculated, making quantitative confirmation of renal phosphate wasting impossible. More importantly, given that the metabolic disturbances underlying this disorder are tightly linked to tumor-derived FGF23, the absence of serum FGF23 and 1,25(OH)_2_D assays further compromises the certainty of etiological diagnosis. Second, although common causes of osteomalacia including nutritional, drug-induced and hereditary factors were systematically ruled out, complete assessments of phosphate excretion function were unavailable, so it cannot be entirely excluded that other single or combined pathogenic factors contributed to the disease. Furthermore, restricted by the hospital’s testing capacity, FGF23 immunohistochemistry and molecular biological examinations of the tumor tissue were not performed, and direct molecular evidence to further verify the causal relationship between D-TGCT and TIO is lacking.

## Conclusion

The diagnostic experience of this case suggests that clinicians should always maintain a high level of vigilance for TIO in patients with refractory or atypical musculoskeletal pain, especially in those suspected of having spondyloarthritis who do not respond to conventional anti-inflammatory treatment. Secondly, attention should be paid to the early screening of characteristic biochemical indicators such as serum phosphorus, and for highly suspicious cases, multi-modal imaging techniques such as ^68^Ga-DOTA-TATE PET/CT should be actively employed for precise tumor localization. Finally, the standardized diagnosis and treatment of TIO rely on multidisciplinary team collaboration involving the Endocrinology Department, Radiology Department, Nuclear Medicine Department, and Pathology Department. In summary, establishing and promoting a rigorous systematic diagnostic process is of significant clinical importance for overcoming diagnostic challenges in such rare diseases, reducing misdiagnosis and mistreatment, and significantly improving long-term patient outcomes.

## Data Availability

The original contributions presented in the study are included in the article/supplementary material. Further inquiries can be directed to the corresponding author.
